# Action leveraging evidence to reduce perinatal mortality and morbidity (ALERT): study protocol for a stepped-wedge cluster-randomised trial in Benin, Malawi, Tanzania and Uganda

**DOI:** 10.1186/s12913-021-07155-z

**Published:** 2021-12-11

**Authors:** Joseph Akuze, Kristi Sidney Annerstedt, Lenka Benova, Effie Chipeta, Jean-Paul Dossou, Mechthild M. Gross, Hussein Kidanto, Bruno Marchal, Helle Mölsted Alvesson, Andrea B. Pembe, Wim van Damme, Peter Waiswa, Claudia Hanson, ALERT Study Team, Gertrude Namazzi, Josephine Babirye, Philip Wanduru, Helle Mölsted Alvesson, Nicola Orsini, Regine Unkels, Virginia Castellano Pleguezuelo, Rian Snijders, Therese Delvaux, Bianca Kandeya, Razak Mussa, Samuel Meja, William Stones, Yesaya Z. Nyirenda, Ahossi Angèle Florence Laure, Antoinette Sognonvi, Armelle Vigan, Banougnin Bolade Hamed, Kéfilath Bello, Christelle Boyi Metogni, Gisele Houngbo, Gottfried Agballa, Hashim Hounkpati, Schadrac Agbla, Joanne Welsh, Muzdalifat Abeid, Tumbwene Mwansisya, Fadhlun M. Alwy Al-Beity, Zamoyoni Julius, Dickson Mkoka, Lilian T. Mselle, Beatrice Mwilike, Helga Naburi, Elizabeth O. Ayebare, Andrea B. Pembe, Ann-Beth Nygaard Moller, Bruno Marchal, Claudia Hanson, Effie Chipeta, Elizabeth Ombeva Ayebare, Hashim Hounkpatin, Pacos Gandaho, Hussein L. Kidanto, Jean-Paul Dossou, Joseph Akuze, Kristi Sidney Annerstedt, Lenka Benova, Lilian Mselle, Mechthild Gross, Peter Waiswa, Wim Van Damme, Jennifer Hall, Erik Lampa, Zahida Qureshi

**Affiliations:** 1 Centre of Excellence for Maternal Newborn and Child Health, Department of Health Policy Planning and Management, School of Public Health, Makerere University, Kampala, Uganda; 2 Department of Epidemiology and Population Health, London School of Hygiene & Tropical Medicine, London, UK; 3 Department of Global Public Health, Karolinska Institutet, Solna, Sweden; 4 Department of Public Health, Institute of Tropical Medicine, Antwerp, Belgium; 5 College of Medicine, The Centre for Reproductive Health, University of Malawi, Blantyre, Malawi; 6 Centre de Recherche en Reproduction Humaine et en Démographie (CERRHUD), Cotonou, Benin; 7 Midwifery Research and Education Unit, Hannover Medical School, Hannover, Germany; 8 Aga Khan University, Medical College, Dar es Salaam, Tanzania; 9 Department of Obstetrics and Gynaecology, Muhimbili University of Health and Allied Sciences, Dar Es Salaam, Tanzania; 10 Department of Disease Control, London School of Hygiene and Tropical Medicine, London, UK; 11 Department of Clinical Nursing, Muhimbili University of Health and Allied Sciences, Dar Es Salaam, Tanzania; 12 Department of Community Health Nursing, Muhimbili University of Health and Allied Sciences, Dar Es Salaam, Tanzania; 13 Department of Paediatrics and Child Health, Muhimbili University of Health and Allied Sciences, Dar Es Salaam, Tanzania; 14 Department of Nursing, Makerere University, Kampala, Uganda; 15 School of Public Health and Community Medicine, Institute of Medicine, University of Gothenburg, Gothenburg, Sweden

**Keywords:** Perinatal health, Maternal health, Intrapartum care, Childbirth, Respectful maternity care, Midwifery, Health system intervention, Sub-Saharan Africa, Hospital

## Abstract

**Background:**

Insufficient reductions in maternal and neonatal deaths and stillbirths in the past decade are a deterrence to achieving the Sustainable Development Goal 3. The majority of deaths occur during the intrapartum and immediate postnatal period. Overcoming the knowledge-do-gap to ensure implementation of known evidence-based interventions during this period has the potential to avert at least 2.5 million deaths in mothers and their offspring annually. This paper describes a study protocol for implementing and evaluating a multi-faceted health care system intervention to strengthen the implementation of evidence-based interventions and responsive care during this crucial period.

**Methods:**

This is a cluster randomised stepped-wedge trial with a nested realist process evaluation across 16 hospitals in Benin, Malawi, Tanzania and Uganda. The ALERT intervention will include four main components: i) end-user participation through narratives of women, families and midwifery providers to ensure co-design of the intervention; ii) competency-based training; iii) quality improvement supported by data from a clinical perinatal e-registry and iv) empowerment and leadership mentoring of maternity unit leaders complemented by district based bi-annual coordination and accountability meetings. The trial’s primary outcome is in-facility perinatal (stillbirths and early neonatal) mortality, in which we expect a 25% reduction. A perinatal e-registry will be implemented to monitor the trial. Our nested realist process evaluation will help to understand what works, for whom, and under which conditions. We will apply a gender lens to explore constraints to the provision of evidence-based care by health workers providing maternity services. An economic evaluation will assess the scalability and cost-effectiveness of ALERT intervention.

**Discussion:**

There is evidence that each of the ALERT intervention components improves health providers’ practices and has modest to moderate effects. We aim to test if the innovative packaging, including addressing specific health systems constraints in these settings, will have a synergistic effect and produce more considerable perinatal mortality reductions.

**Trial registration:**

Pan African Clinical Trial Registry (www.pactr.org): PACTR202006793783148. Registered on 17th June 2020.

**Supplementary Information:**

The online version contains supplementary material available at 10.1186/s12913-021-07155-z.

Contributions to the literature

- Single component facility-based interventions to improve quality of care have modest to moderate effects. It is unknown if carefully designed, multi-component interventions can lead to greater effects with consistent implementation of evidence-based practices.
- Within the debate to redesign perinatal care in low- and middle-income countries for quality and equity, our research in hospital settings, how they function and how they can improve processes is of utmost relevance for the global ambition to provide respectful and safe perinatal hospital care for all.
- Our intervention design includes end-user participation and applies a health system lens to increase the intervention’s relevance to initiate and support effective and context-sensitive processes and sustainable improvements.
- We will explicitly merge competency based training and quality improvement into an integrated approach supported by cascade mentoring and leadership training.
- New data systems are needed to better understand the drivers of ill-health and mortality. We will test the feasibility of a perinatal e-registry in selected hospitals.

## Background

There are two million stillbirths globally, and 2.4 million newborns die before reaching one month of age every year [1, 2]. Almost 300,000 women die during pregnancy and childbirth annually [3]. Evidence-based care during the intrapartum period, from the onset of labour to the expulsion of the placenta, carries the greatest lifesaving potential [4]. The importance to address hypoxic-ischaemic insults causing long-term disabilities or perinatal death is increasingly highlighted [5]. Moreover, this period provides an opportunity to prevent 800,000 malnutrition-related child deaths annually by initiating breastfeeding [6]. It is considered a central hub for referral and communication along the continuum of care linking antenatal, postnatal and child health care [7].

Clear evidence-based guidelines for the provision of routine and emergency care during the intrapartum period are established [8–10]. However, evidence suggests that insufficient provider competencies and substandard professional norms rooted in inadequate pre-service training and malfunctioning processes and operations constrain the implementation of such guidelines for maternal and newborn health [11]. Mistreatment of women is also increasingly highlighted as a major challenge during the intrapartum period [12].

Quality improvement (QI) and training are proven to reduce mortality [13, 14]. A recent study from Uganda and Kenya found that combining training and QI was a successful strategy to achieve more considerable perinatal mortality reductions [13]. Two recent reviews concluded that multi-component strategies addressing several underlying factors related to inadequate care have a larger effect on improving health providers’ practices compared to single component strategies [15, 16]. Therefore, there is a need to test the effectiveness and cost-effectiveness of a multi-component intervention. Further, it is critical to understand what can work in different contexts and if it works, why, through assessing acceptability, adoption, appropriateness, and feasibility of an intervention [14, 17].

**Fig. 1:**
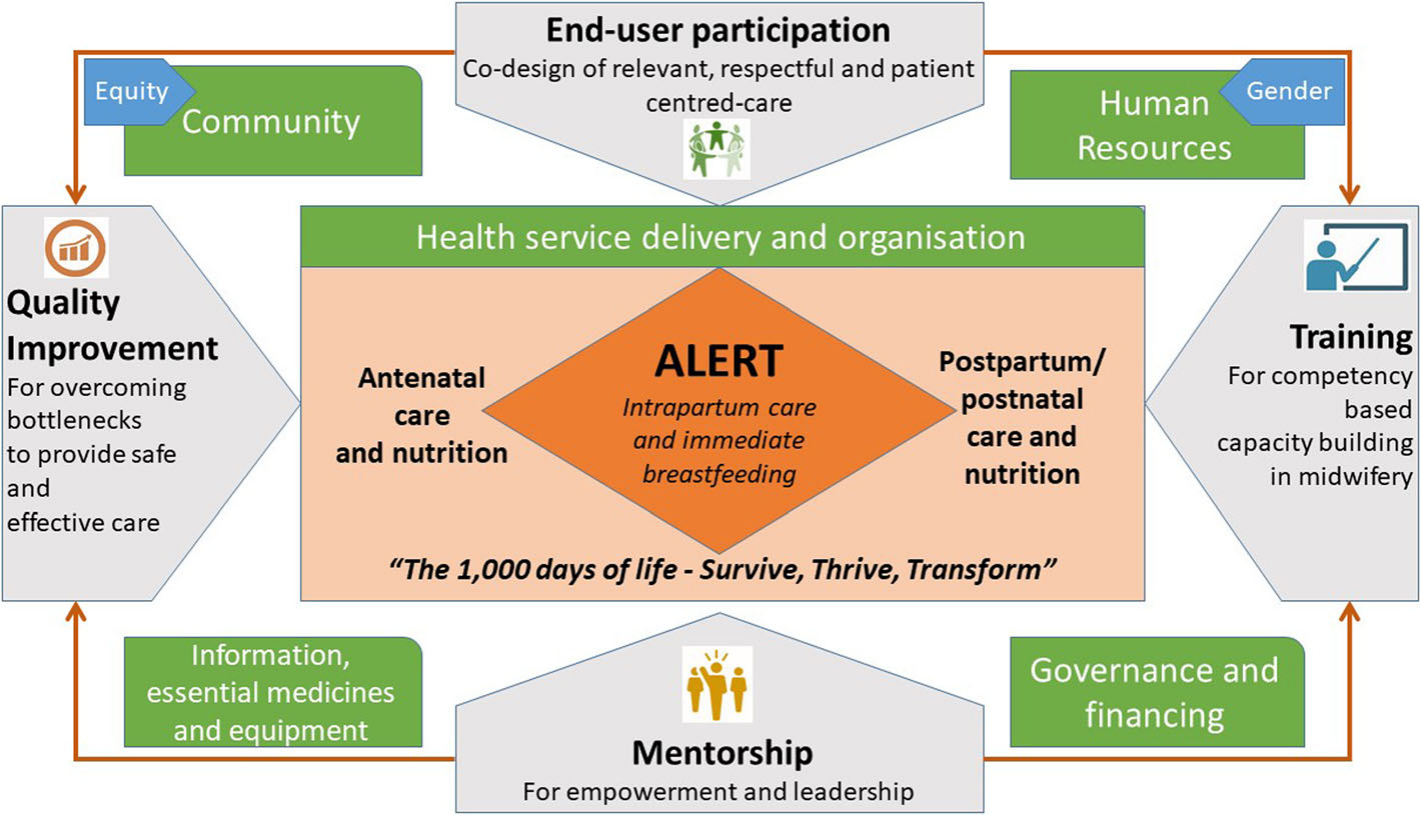
ALERT Conceptual Framework

In response, we propose developing and evaluating a comprehensive and multilevel intervention termed **A**ction **L**everaging **E**vidence to **r**educe perinatal Mor**t**ality and morbidity in sub-Saharan Africa (ALERT). ALERT will focus on intrapartum care and midwifery with a health care system strengthening lens. ALERT specifically targets hospital maternity units and will include i) end-user participation of women, families, and midwifery providers to co-design the intervention; ii) in-service midwifery competency-based training; iii) empowerment and leadership mentoring of maternity unit leaders, and iv) QI in the maternity ward, supported by district-based bi-annual coordination and accountability meetings (Fig. 1).

Our theory of change can be summarised as follows. End-user participation built around narratives of women, families and midwifery providers is expected to contribute to embed responsiveness and mutual respect in the training and QI. This should lead to improvements in effective communication, respect, dignity and emotional support. Together with the training and mentoring, the co-design is expected to lead to more competent and motivated midwifery providers, thus providing improved care (e.g., improved foetal monitoring, timely decisions, and emergency and client-centred support during labour). In addition, immediate breastfeeding, encouraged for all women - including those experiencing caesarean sections – contributes to improved bonding, nutrition and optimal growth and development in early childhood. Finally, the QI enhances the use of data to make information systems more actionable. QI and the district coordination and accountability mechanism should improve resource allocation efficiency (Fig. 1).

The specific study objectives are:

1. To assess the ALERT intervention’s impact in hospital maternity units on perinatal and maternal health outcomes, including women’s experience of care.
2. To evaluate the process of implementation of the intervention to understand what works for whom and under what situation.
3. To conduct a cost-effectiveness analysis of the ALERT intervention.

We hypothesise that ALERT will i) reduce in-facility early perinatal mortality; ii) reduce perinatal and maternal morbidity; iii) improve evidence-based practices (immediate breastfeeding, experience of care); iv) strengthen communication links between primary care and hospitals as well as ante-, intra-, postnatal and child health care; and v) strengthen professional exchange networks through mentoring for sustained learning and action.

## Methods: description

### Study design

We will use a stepped-wedge cluster-randomised design with a nested process evaluation based on realist evaluation [18] to evaluate the process of implementation of ALERT to understand what works for whom and under what conditions [19]. We will also conduct a cost-effectiveness analysis to inform scalability. The cluster design was chosen as the intervention will be delivered at the hospital level. Our clusters are defined as a maternity ward of a hospital offering caesarean section and blood transfusion services with a minimum caseload of 2500 births per year. A stepped-wedge design was chosen to mirror scale-up for policy buy-in and for statistical efficiency as we expect larger cluster-level differences [20]. In addition, it enables the realist process evaluation and economic evaluation to take place in hospitals where we expect the intervention to be sufficiently mature in the way it is implemented. This protocol follows CONsolidated Standards of Reporting Trials (CONSORT) for stepped-wedge cluster randomised trial (SW-CRT) (Additional file 1) and the Standards for Reporting Implementation Studies (StaRI) (Additional file 2).

### Context

ALERT will be implemented in four hospitals in Benin, Malawi, Tanzania and Uganda. These countries were purposely selected to allow for a range of health system characteristics and implementation challenges. While Malawi, Tanzania and Uganda share many health system characteristics (strong public health structures, nurse-midwifery and non-direct entry into midwifery education), there are also distinct differences (Table 1). For example, Malawi and Tanzania have strong task-shifting policies in maternity care whereby mostly non-physician clinicians perform caesarean sections [22]. In Uganda and Benin, in contrast, caesarean sections are performed exclusively by medical doctors. In Benin, direct entry into midwifery education is practised and maternity care is thus largely provided by midwives.

**Table 1:** Characteristics of study countries

	Benin	Malawi	Tanzania	Uganda
**Country-level indicators**				
Estimated population (in 2020, million)	12.1	20.3	62.8	47.2
Maternal mortality ratio per 100,000 live births (2017) [3]	397	349	524	375
Neonatal mortality rate per 1000 live births (2019) [1]	31	20	20	20
Stillbirth rate per 1000 total births (2019) [2]	20.3	16.3	18.8	17.8
% of live births in health facilities #	83.9%	91.4%	62.6%	73.4%
% of facility births in hospitals ##	35.4%	42.2%	47.8%	47.8%
Annual growth rate in % of births in health facilities (most recent DHS compared to survey between 2004 and 2006 ##	0.7%	2.5%	2.6%	5.8%
% of all live births by CS # - Poorest v Richest wealth quintile	5.1 1.6–12.3%	6.1 3.0–9.1%	5.9 2.4–15.8%	6.2 2.7–14.2%
**Among live births in health facilities**				
% checked before discharge after facility births ##	81%	57%	51%	47%
% of all live births by CS#	6.1%	6.3%	9.5%	8.3%
**Among live births in hospitals**				
Neonatal mortality per 1000 live births ##	32.8	35.8	31.8	27.1
% of newborns breastfed within 1 h of birth ##	61.8%	73.1%	54.9%	65.3%
**Health system indicators**				
Doctors /10,000 people population ^	0.8 (2018)	0.4 (2018)	0.1 (2016)	1.7 (2017)
Nursing cadres /10,000 people population^	3.9 (2018)	4.3 (2018)	5.8 (2017)	12.4 (2018)
Predominant midwifery provider [21]	Midwife	Nurse-midwife	Nurse-midwife	Midwife
Hospital beds / 10,000 people population^	5 (2010)	13 (2011)	7 (2010)	5 (2010)
Current health expenditure per capita (USD PPP, 2018)^	83.2	119.5	112.5	139.3
Out-of-pocket expenditure as % of current health expenditure (201^	45	11	24	38
User fees for childbirth (vaginal/caesarean)^	Official fees	No official	No official	No official

*CS* caesarean section

# DHS StatCompiler and Survey reports for Demographic and Health Survey data, Benin; 2017–8; Malawi: 2015–16; Tanzania: 2015–16; Uganda: 2016

## Additional analysis of Demographic and Health Survey data, Benin; 2017–8; Malawi: 2015–16; Tanzania: 2015–16; Uganda: 2016

^WHO observer http://apps.who.int/gho/data/node.main.HWFGRP_0020?lang=en

### Targeted sites and participants

**Fig. 2:**
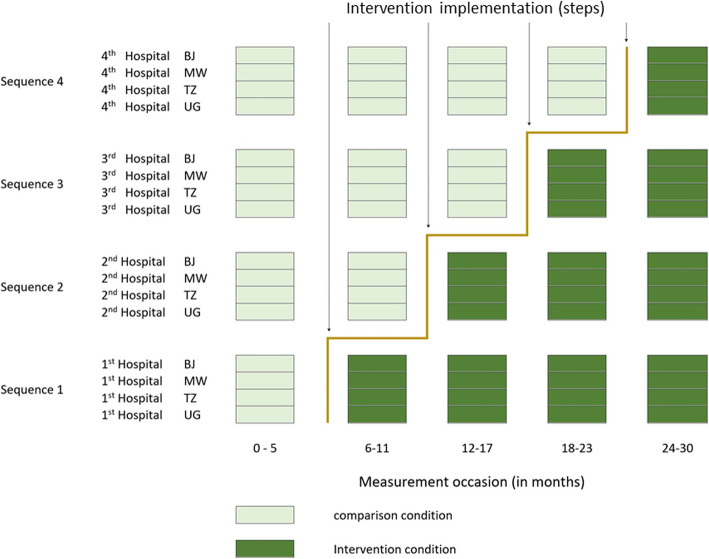
ALERT intervention implementation schematic. Light green indicates the comparison cluster. Dark green indicates the cluster is receiving the intervention. BJ: Benin, MW: Malawi, TZ: Tanzania, UG: Uganda

**Fig. 3:**
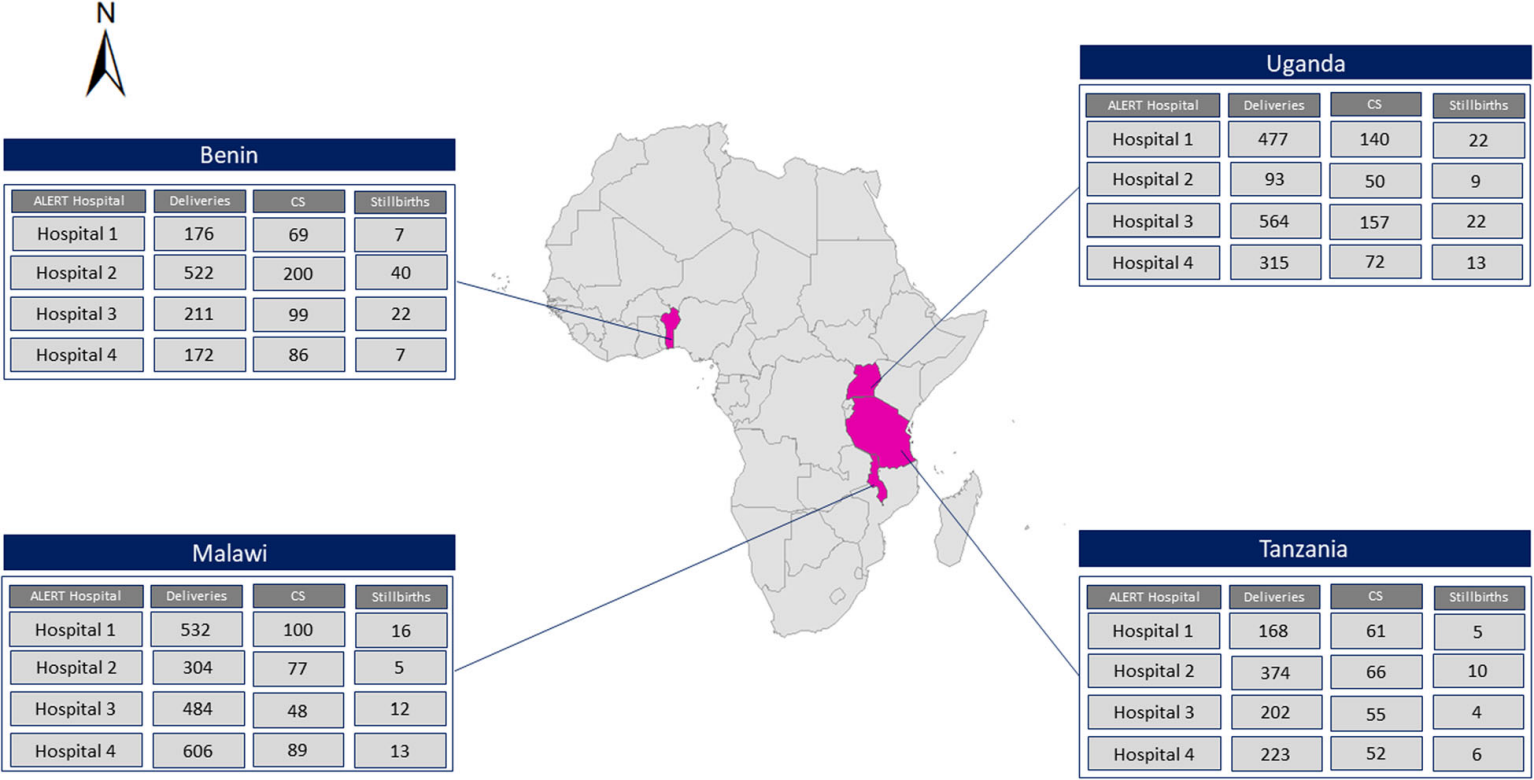
Map of the ALERT countries with key indicators for the selected study hospitals. CS: Caesarean section

The trial will commence April 2021 for 30 months (Fig. 2). Trial hospitals were selected purposely to reflect the range of facilities and include typical hospitals currently caring for 30–50% of all births for the respective country [23]. In March 2020, we consulted with national Ministries of Health and prepared a list of all hospitals meeting the selection criteria of i) minimum caseload of 2500 births per year required based on trial sample size calculation; ii) caesarean section and blood transfusion services available; iii) preferably located in rural districts; and iv) consisting of a mix of typical public but also private-not-for-profit (faith-based) hospitals. We included public and private-not-for-profit hospitals to reflect the typical landscape of hospitals in sub-Saharan Africa and improve our results’ generalizability. We then selected four hospitals in each country (Fig. 3).

The intervention directly targets health care providers involved in intrapartum care and all women who give birth in the participating hospitals during the study period. In this study, the term ‘maternity care providers’ refers to nurses, nurse-midwives, midwives, auxiliary staff and medically trained staff such as obstetricians working in the maternity ward at one of the study facilities. Women will be eligible if they give birth to a newborn weighing ≥1000 g, which is a proxy for viable gestational age in settings with poor gestational age measurement. Women who gave birth in another location but receive care in the hospital after childbirth will not be included in the study as our intervention targets the intrapartum period.

### The intervention

Intervention development was conceived in response to the SC1-BHC-19-2019 call from the European Commission to innovate and evaluate interventions to bridge the knowledge-do gap to improve health during the first 1000 days of life. Further, our intervention links to the 2030 Sustainable Development Goal agenda [24] and the Survive, Thrive, Transform aspirations of the United Nations [25].

The ALERT intervention focuses explicitly on the key elements of intrapartum care of i) admission, labour monitoring; ii) immediate maternal and newborn care; and iii) readiness and care for complications (Additional file 3). Thus, ALERT will cover all stages of labour, biologically effective interventions (such as appropriate admission, foetal monitoring, emergency preparedness like reducing time from decision to perform a caesarean section) and improving experience of care (such as promoting companionship and communication in the maternity wards).

Our intervention is based on previous research in conceptualising and evaluating care QI and training interventions [14, 26, 27] and learning from the large Safe Childbirth Checklist trial in India [28]. Key intervention elements are continuous training and QI based on the assumption that the combination of these two is needed to address the underlying causes of inconsistent implementation of evidence-based practices.

Further, intervention development and adaptation rely on end-user participation to consider women, families, and health providers’ perspectives [29]. The design pays attention to the experience of interaction between people and health systems. Understanding health systems responsiveness offers an opportunity to adapt care to changing clients/patients’ needs, promote women’s access to effective interventions and improve the quality of health services, ultimately leading to better health outcomes [30].

The intervention will include several training modules based on competency-based methodology and using the Laerdal Global health Mama Birthie low-cost models [31]. The training will be made available to maternity providers, similar to the successful Helping Mothers and Babies Survive modules [27].

Mentorship is increasingly recognised as an effective strategy to improve healthcare quality, either as part of QI bundles or as a stand-alone intervention [32, 33]. The ALERT mentoring and leadership training intervention component will use a cascade approach with i) in-facility clinical mentors linked to the QI approach and training; ii) mentorship from in-country ALERT staff for the head of the maternity unit; and iii) mentoring within the international ALERT team. Mentoring will address individual professional attitudes, inter-professional collaboration (teamwork), leadership strengthening for resource negotiations, and other aspects.

The QI intervention aims to i) support the consistent implementation of the trainings provided; ii) address operational deficiencies identified during the formative research as part of the end-user participation strategies; and iii) support linkages between established maternal death review teams as well as other hospital improvement structures. We will use standard Plan-Do-Study-Act methodology. Data for follow-up will come from the perinatal e-registry or registers adapted to the type of data.

### Implementation strategy

The intervention will be delivered by maternity care providers in the study hospitals and supported by our research teams who are based in national universities and well-placed to deliver training and engage with supporting QI approaches. Local hospital-based training and management resources will be mobilized and integrated. To support the ALERT intervention’s institutionalisation and sustainability, there must be strong leadership from the districts and collaboration with the Ministries of Health, training institutions, and integration into existing QI structures in each country. We further linked our training approach to training resources within the countries, thus trainers of trainers as available at national and subnational level.

In line with Juran’s trilogy and the WHO, we concur that promoting the combination of quality planning, control and improvement allows for more sustainable interventions [34]. The QI intervention will be informed by the collaborative QI approach [35] and will explicitly link to QI approaches already implemented in the facilities. To bolster knowledge, an adapted QI refresher training will be provided including the PDSA and problem-solving methods. Bottlenecks identified during the health facility assessment, operational deficiencies identified during the ALERT competency-based training sessions, and recommendations arising from the maternal death reviews will be the target of PDSA cycles addressed by the QI team. The hospital-based QI team will be supported by our research team and the head of the maternity unit to develop and implement feasible, small scale solutions.

We recognise the barriers described to consistent implementation of QI particularly in resource-poor and understaffed settings [14, 36, 37]. PDSA cycles, although widely used, have been associated with limited effects [26, 38]. With this in mind, we plan to make adaptations to the collaborative QI approach in order to increase the effectiveness of the ALERT QI package (see Table 2 in additional file 3). By explicitly linking to the existing QI structures including perinatal audit and management, we aim to ease implementation and improve potential scalability [41, 42]. The mentoring approach linking to central national institutions is expected to improve accountability to support the structured and regular implementation of QI and thereby the needed *control* aspect as well as the link to the local management structures. The end-user participation element of the intervention design will allow the incorporation of quality planning which the WHO is now proposing as an essential component of QI [38].

**Table 2:** Primary and secondary outcome indicators

Primary outcome indicators	Definition	Methods to obtain outcome
Fresh Stillbirth rate	Number of fresh^a^ stillbirths of at least 1000 g expressed per 1000 live and stillbirths	Perinatal e-registry
In-facility early perinatal mortality	Number of fresh stillbirths (as above) and up-to discharge neonatal deaths per 1000 live and stillbirths (composite indicator)	Perinatal e-registry
**Secondary outcomes**		
Hypoxic-ischaemic event rate	No of neonates with APGAR < 7 at 5 min per 1000 live and stillbirths	Perinatal e-registry
Hypoxic-ischaemic event rate	Umbilical cord lactate of > 5.5 mmol^b^ per 1000 live and stillbirths	Perinatal e-registry, (sub-sample)
Neonatal seizures	No of neonates diagnosed with seizures per 1000 live and stillbirths	Perinatal e-registry
Caesarean section rate (%)	No of caesarean section per 100 live and stillbirths	Perinatal e-registry
Severe maternal morbidity	No of women with morbidities^c^ per 1000 live and stillbirths	Perinatal e-registry
Responsiveness (%)	Validated questionnaire [39] (% score) per 100 live and stillbirths	Survey among women at discharge (exit interviews)
Mistreatment (%)	Proportion of women reporting mistreatment per 100 live and stillbirths	Survey among women at discharge (exit interviews)
**Process indicators (selected)**		
Detection of foetal distress	No. of detected foetal distress events per 100 deliveries defined by FIGO [40]	Perinatal e-registry
Decision-to-birth time for caesarean section	Median time (minutes) between decision to do a caesarean section to the birth of the baby	Perinatal e-registry

^a^Fresh stillbirth is defined a stillbirth that happened during labour at the respective facility, thus where the foetal heartbeat was positive at admission; ^b^The cut-off level may be revised based on data from an ongoing study in Uganda and validation work; ^c^Severe maternal morbidity will be defined using pragmatic criteria of major interventions (hysterectomy, laparotomy, blood transfusion, admission to intensive care unit or referral to higher level facility)

### Methods: evaluations

This study includes three evaluations; 1) stepped-wedge trial; 2) realist process evaluation and 3) economic evaluation. The methods for each are described below.

### Stepped-wedge trial

#### Outcomes

Our primary outcome is in-facility early perinatal mortality defined as in-facility (fresh) stillbirth and 24-h neonatal mortality. Selected secondary and process outcomes are listed in Table 2. For a sub-sample of births, we will use lactate measurement using a simple point-of-care test (Nova Biomedical, StatStrip Xpress-I lactate) to obtain an objective measurement of hypoxic-ischaemic insults to be used in conjunction with the more subjective APGAR score due to interrater differences. It is suggested that lactate provides good predictive values on hypoxic-ischaemic insults as conventional pH measurement and base excess [43, 44]. Breastfeeding initiation will be assessed using information recorded in the perinatal e-registry and women’s reports at the time of discharge will be integrated into the exit interviews to determine responsiveness and experience of mistreatment.

#### Method of data collection

The primary and secondary outcome data will be collected through a perinatal e-registry and exit interviews with women being discharged following childbirth. The perinatal e-registry will include standard indicators of pregnancy risks and care received during the antenatal and perinatal period. The indicators were informed by similar clinical data collection in the European Union [45] and Tanzania [46]. We will support standardised admission and follow-up case notes to improve continuous documentation during care provision. After short training sessions facilitated by research staff, data will be entered continuously in the maternity ward by midwifery staff or data clerks (based on country preference). Exit interviews will be administered by research staff every six months during the implementation period (six time points) to 50 randomly selected women who had given birth in each hospital. To assess responsiveness and mistreatment, we will use a recently validated questionnaire with some adaptations [47].

#### Data management

The ALERT perinatal e-registry will provide primary and some secondary outcomes and will be implemented in all study hospitals. All women who meet the eligibility criteria will be included. The perinatal e-registry data will be entered on the maternity ward using the Research Electronic Data Capture (REDCap) platform available on tablets or computers [48]. The programme will have inbuilt ranges and branching logic programmed to improve data quality. Monthly data checking and feedback to providers will also be implemented. Weekly paper-based summary sheets will be used to check data completeness, and double-entry of data for 10% of the records will be done by an external facility supervisor. Supervision structures will include in-hospital supervision by an external resource to the maternity ward and by an ALERT research team data manager. 

#### Sample size

The Hemming et al. formula for stepped-wedge trials was used to calculate the study’s power [49]. We used intra-cluster correlation coefficients for stillbirth and neonatal mortality from a study in Malawi [50] and maternal morbidity from a recent trial [51]. The inclusion of 16 hospitals, each with at least 2500 births per year, will give sufficient power (75–80%) to detect a 25% reduction among in-facility early perinatal mortality with baseline rates between 1.4 to 2.0% and 95%-confidence intervals. We also have sufficient power to assess several secondary outcomes, including maternal morbidity (Additional file 4).

#### Randomisation

Randomisation will be stratified by country to ensure that hospitals are randomly selected and enrolled in six-monthly steps (four hospitals in four steps) in each country. Randomisation was performed by a statistician, independent from the implementation team, once the hospitals had consented to participate in the study. As with all training and QI interventions, we cannot blind participants (hospitals) to the intervention. However, women and families might not be aware of the exact step in the implementation of ALERT at the hospital where they give birth.

#### Statistical methods

The statistical analysis will be “intention-to-treat”, comparing ALERT intervention clusters (hospital maternity wards) with comparison clusters where care is provided according to national standards. We will define a “transition” period of two weeks during which the intervention is provided and adopted by the respective hospitals.

We will use descriptive analysis to review the trends using interrupted time series analysis from the 30 months of data collection through the perinatal e-registry [52]. Seasonal variations will be described (e.g. birth weight and neonatal mortality) [53, 54]. While secular declines in stillbirths and early neonatal mortality have been slow in the past; we expect annual declines of at least 2% [55]. We will review secular trends over strata (countries) and clusters (hospitals) and estimate the heterogeneity of the effects across clusters as advised by Hemming et al. [56]

Considering the limited number of clusters, we will use generalised estimating equations (GEE) adjusting for clusters and for the small sample [57]. We will adjust for clustering, time-trends and the sequence of inclusion of hospitals [56] and other methods to perform small-sample adjustments [58].

#### Sub-group analysis

Additional sub-group analysis by stratification on select covariates such as birth weight, mode of delivery, time of delivery and type of outcomes will be conducted based on the power and sample size plausibility.

#### The realist process evaluation

**Fig. 4:**
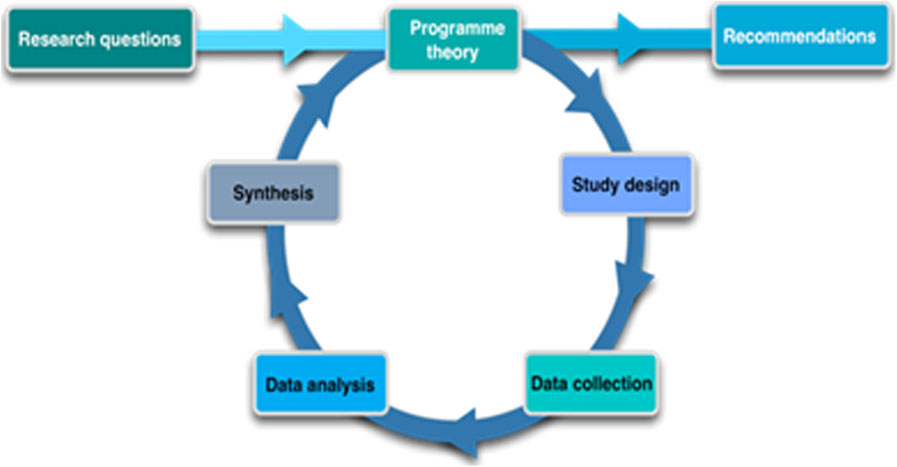
The realist research cycle [59]

We will assess how the intervention works by assessing how actors take up and implement the intervention components based on mechanisms that are triggered in specific contexts to generate the outcomes. We will analyse the differential effects of interventions in the ALERT settings: why is an intervention successful in one setting but perhaps without effect in another? The evaluation is structured along the realist research cycle [59], which starts with the development of an initial programme theory (Fig. 4).

The initial programme theory will be developed based on the ALERT theory of change, a review of the most current literature and discussions with ALERT researchers. We will adopt a multiple embedded case study design to test the initial theory. In each country, we will select one hospital where the ALERT intervention is implemented at step 1 of the stepped wedge design and a second hospital involved in step 3. This phased recruitment of facilities will enable us to assess how the length of exposure to the intervention and changes over time influence observed outcomes. Selecting two hospitals per country will allow for cross-case comparison and identifying how mechanisms play out differently in different hospital-specific contexts. In each hospital, data will be collected on the implementation of the intervention, the context, the actors and the processes triggered by the intervention through a document review and interviews with (1) hospital managers, heads of maternity, midwives and district directors of health, and (2) mothers and families. We will also draw upon data from other work packages and participatory reflection sessions at ALERT consortium meetings. Audio-recordings will be done on devices which allow data encryption and in case this is not possible, recordings will be immediately transferred to a computer where data can be encrypted. All recordings will be verbatim transcribed and translated to English where needed. All transcripts will be entered in a NVIVO database and pseudo-anonymized to the highest degree possible using identifiers and codes for key variables. The ICAMO heuristic will be used in the data analysis [60]. The data will first be categorised using the intervention-actor-context-mechanism-outcome configuration. Next, a retroduction approach will be adopted, whereby explanations for the observed outcomes are identified by looking into the mechanisms, actual intervention modalities, actors and context elements. In-case and cross-case analysis will allow for the formulation of the ‘final’ programme theory, which will indicate what it is about the ALERT intervention that works for whom and in which circumstances. This will inform recommendations for scaling up the intervention and tailoring it to different contexts.

#### The economic evaluation

Closely linked with the realist and effect evaluation will be an economic evaluation of the ALERT intervention. According to Drummond et al., economic evaluation is defined as “the comparative analysis of alternative courses of action in terms of both their costs and consequences” [61]. We propose to evaluate the economic impact of the ALERT intervention by conducting cost-effectiveness analysis, focusing on the mature intervention implemented in step three hospitals. The incremental cost-effectiveness ratio (ICER) will focus on the net costs per one reduction in stillbirth and in-facility perinatal mortality. We will utilise the Consolidated Health Economic Evaluation Reporting Standards (CHEERS) to optimise the reporting of our evaluation [62].

#### Dissemination

We will follow the European Union’s open-access policy and strive to make all ALERT training modules, reports, and scientific articles publicly accessible. We will utilise the following dissemination channels: leaflets, a website (alert.ki.se), workshops and meetings at the local (district) and national level, conference presentations (local, national, and international), and peer-reviewed publications and reports. Key stakeholders, including the study participants (i.e., end-users), Ministry of Health policymakers, and other stakeholders interested in improving maternal and child health will be proactively sought out, and findings from the study shared.

## Discussion

### Innovation and potential impact

We will test an innovative multi-component intervention that was carefully conceptualised based on previous research [13, 14], which will be further refined based on the formative research. The co-design component recognises the need for continuous adaptations of QI, and we believe this is the first QI initiative in this field explicitly integrating formal end-user participation. The ALERT intervention also uses system thinking principles. In several settings across sub-Saharan Africa, interventions and policies to implement (and mainly maintain) the delivery of quality maternity care show inconsistent results. Quality care challenges are increasingly conceptualized as system-related complex problems.

Our study applies systems thinking by adopting a theory of change approach and implementing a process evaluation that is based on realist evaluation. It not only starts its empirical research from existing knowledge and theories but will also contribute to develop better theories on implementing QI initiatives in maternal health. Furthermore, the realist process evaluation allows for exploring and assessing the complex causal processes underlying the observed outcomes and identifying the required context factors. In that sense, it demonstrates how theory can be used in the three ways described by Nilsen (2015) [63] (1): to explain how implementation outcomes are shaped (2), to provide a solid foundation for evaluation of the implementation of interventions and (3) to assess and inform the translation of research findings into policy and practice.

High maternal and perinatal mortality rates and the large increases in the proportion of childbirths occurring in health facilities make it paramount to identify and evaluate interventions aimed at reducing mortality during birth, especially since relatively simple, cheap and effective evidence-based interventions are available. While there is good evidence that single component interventions improve health workers’ practices and health outcomes, our hypothesis is that our innovative ALERT package will work synergistically and lead to larger and sustainable reduction in in-facility mortality and morbidity. The element of end-user participation explicitly addresses the need to adapt QI to the needs in the settings [14]. Our attention to leadership and mentoring responds to the findings that more holistic approaches are likely to lead to more ownership and sustainable changes [36].

We will work in hospitals that operate under resource-limited conditions. Too few midwifery providers care for a growing number of women. The lack of professional midwives is increasingly acknowledged globally [64]. Various pathways in the ALERT intervention aim to strengthen the quality of intrapartum care and midwifery with a view on an enabling environment and improved knowledge management.

In recent years, many resources were committed to improving access to facility-based births to achieve the Millennium Development Goal 5 and now the SDGs – with major success [65]. In view of these developments, quality of care in these facilities needs to be prioritised. We believe that quality of care and the resources available for maternity care must improve as hospitals care for an ever-increasing proportion of births and emergency referrals. Through stakeholder engagement, we anticipate that during the implementation of ALERT, resources available to hospitals, such as staff allocation, investment in physical infrastructure, medicines and medical supplies and other resources will increase. The leadership mentoring and quality of care improvement components of ALERT might contribute to this increase in resources.

Data collection will take place during the SARS-CoV-2 pandemic which impacts all kinds of daily life. This raises the need for innovative adjustments in terms of learning resources and training. As our protocol is affected by the COVID-19 pandemic, we are committed to work on resilient solutions for a post-pandemic situation. We will add knowledge to this new situation with appropriate digitalised technologies with regard to communication, teaching and evaluation.

Rigorous evaluations of multi-component interventions are needed and will add to the existing body of literature on what combination of components are best suited to improve intrapartum quality of care [13]. Since ALERT is a multi-faceted intervention, each of the components will have its own set of implementation strengths and challenges. There is a need to contextualize each component and an opportunity to learn from a feasibility and acceptability perspective within a multi-country setting. Highlighting the necessity of measuring what works where and understanding the acceptability of each of the components in the different contexts.

### Methodological considerations

There are some important methodological considerations for the ALERT trial. Additional variables related to COVID-19 were incorporated in the data collection tools after the initial ethics submission and approved by all institutional review boards as an amendment. The intervention development will consider the changed realities of providing care during the COVID-19 pandemic and post-pandemic period.

One could argue that the generalisability of the results of the stepped wedge study may be limited due to the small number of health facilities and countries included in the study. Furthermore, there may be some selection bias, as the study hospitals are large high-level hospitals that tend to have more high-risk, complicated pregnancies and births. However, because of its comparative design, its building upon existing evidence and theory, and its attention to context, the realist process evaluation’s empirical research will provide insights in the conditions that facilitate or inhibit the ALERT intervention, thus providing relevant information to policymakers in other countries.

Neonatal deaths and some important exposure variables such as gestational age are prone to reporting bias as ultrasound in early pregnancy is not routinely used. While we have designed our perinatal e-registry carefully, challenges in completeness and quality of documentation will need to be considered. Exit interviews to assess responsiveness and mistreatment are challenging as women might feel pressured to hide negative feelings and there are social risks in complaining. Differences in reported negative experiences between facility-based and community-based assessments have been reported [66]. However, funding limitations do not allow us to conduct follow-up visits at home. We will carefully train interviewers to limit the social desirability bias.

Stepped-wedge designs are particularly susceptible to secular changes in the main outcome. Therefore, we will allow for a longer assessment period than a traditional parallel-group trial [67]. However, the ALERT trial is implementing an perinatal e-registry to measure the outcome variable continuously, thus minimizing this challenge. We also plan to examine time trends and seasonality and identify the appropriate method to adjust for this. Finally, over the trial 30-month period, other interventions might occur in the hospitals, making it difficult to disentangle the effect of ALERT.

We believe our comprehensive evaluation using qualitative and quantitative methods will provide important information on the functioning and effects of QI in typical hospitals in sub-Saharan Africa and will further contribute to the evolving literature on the effects and processes of QI in maternal and new-born care.

### Trial status

Not yet recruiting.

## Supplementary Information

**Additional file 1.** CONsolidated Standards of Reporting Trials (CONSORT) for stepped-wedge cluster randomised trial (SW-CRT).

**Additional file 2.** Standards for Reporting Implementation Studies (StaRI).

**Additional file 3.** ALERT intervention description.

**Additional file 4.** Sample size calculation (ICC, Inter Cluster Coefficient).

## Data Availability

Quantitative datasets will be cleaned and made openly available in an open access online repository (Zenodo - https://www.zenodo.org/), after anonymisation alongside with meta-data, to facilitate data sharing and re-use. The training materials created during ALERT, publications, reports will be freely available on the ALERT website and disseminated through the ALERT networks. Qualitative data will not be made available to researchers outside the partner institutions. This decision was approved by the EU and is mainly based on three principles: 1) qualitative data are difficult to interpret or reuse without extensive knowledge of the context and how it was originally collected (i.e. experience and background of the data collector), 2) with the small number of hospitals included in the project it would be difficult to guarantee participants complete anonymity, and 3) the quality of the response may differ if participants knew their spoken word would be made available in an open repository.

## Abbreviations

ALERT: **A**ction **L**everaging **E**vidence to **R**educe perinatal mor**T**ality and morbidity
CEA: Cost-effectiveness analysis
ICER: Incremental cost-effectiveness ratio
QI: Quality Improvement
SDGs: Sustainable Development Goals
WHO: World Health Organization
