# Supplementary material for: Action leveraging evidence to reduce perinatal mortality and morbidity (ALERT): study protocol for a stepped-wedge cluster-randomised trial in Benin, Malawi, Tanzania and Uganda
Source: BMC Health Serv Res. 2021 Dec 11;21:1324. doi: 10.1186/s12913-021-07155-z (PMC8665312; doi:10.1186/s12913-021-07155-z)
Supplement: Supplementary file 3 — Additional file 3. ALERT intervention description. [file 12913_2021_7155_MOESM3_ESM.docx]

**Additional File 3:**

**ALERT INTERVENTION DESCRIPTION**

# Background

The ALERT project aims to use end-user participation to tailor a comprehensive intervention to local needs. The intervention will be comprised of competency-based training for midwifery care providers, leadership mentorship, and quality improvement. Combined, these interventions aim to reduce perinatal morbidity and mortality in the four ALERT countries. The intervention was conceptualised taking two recent reviews into consideration which concluded that multi-method strategies addressing multiple underlying reasons for inadequate care have a larger effect on improving health provider’s adherence to good practices than single method strategies.^1,2^ A recently published study from Uganda and Kenya further provides evidence that packaging training together with quality improvement maybe a successful strategy to achieve larger reductions in perinatal mortality.^3^ Our work is also taking learning from evaluating quality improvement projects in Tanzania ^4,5^ and in India.^6^

The intervention is focusing on medium to large hospital maternity wards. This choice has been made in view that hospital-based maternities are providing childbirth care for an increasing number of births in Africa. Also, there is much attention to close the quality-gap for maternal and newborn health, ^7^ and facilities with higher caseload and access to emergency obstetric care are better placed to deliver quality maternal and newborn care.^8^

The intervention will focus primarily on maternity care providers defined as nurses, nurse-midwives, midwives, auxiliary staff and medically trained staff such as obstetricians working in the maternity ward at one of the study facilities. Through our health system lens, we will further link to district and hospital managers and other structures supporting quality perinatal care.

The intervention was developed by a research team (the ALERT team) comprised of 1) midwives, obstetricians and nursing cadres – all with education and training experience, 2) health systems researchers, and 3) implementation science experts.

The intervention components are developed based on formative research, and include:

- ***National context and health facility assessment*** – to understand the organisational and governance structures of the hospital that affect its overall functional status.
- ***Interviews with women*** – to understand women’s perspectives of the care they receive and to identify any gaps in care provision.
- ***Interviews with midwifery care providers*** ­- to generate insights around childbirth and the maternity ward setting from the perspective of midwifery care providers.
- ***Focus group discussions and/or natural group discussions with companions*** – to generate insights into the experiences of interacting with midwifery care providers from the perspective of those acting as women’s companions during the process of labour and birth.
- ***Go along methodology with mothers and shadowing of midwifery care providers*** –observation of behaviour, communication and interactions between midwifery care providers and women to better understand individual and environmental constraints impacting care.
- ***Midwifery care provider self-administered questionnaire*** – to identify knowledge levels related to intrapartum care.
- ***Skills drills sessions with midwifery care providers*** –to gain an insight into the skills and competence of midwifery care providers as they provide intrapartum care.

Natural group discussions with midwifery care providers following analysis of data collected in the formative research phase will allow priority areas of focus to be presented. Midwifery care providers will then discuss and identify “change ideas” which can be used to support improved adherence to evidence based practice in these areas.

# Initial theory of change and synergies between components

The intervention with its four components is thought to act synergistically. This is represented in the initial Theory of Change for the intervention (Fig 1). In this theory, four main components interact, namely i) end-user participation, ii) quality improvement (QI), iii) training of midwifery care providers, iv) mentorship of maternity unit leaders. The synergies between the main four components include: end-user participation ensures that the needs of women, families and midwifery providers are realised and reflected in the training and QI (Fig 2). Better trained and supported midwifery providers will provide better care including improved foetal monitoring, emergency and client-centred support during labour. Better skilled and empowered midwifery providers will also be better able to make faster decisions, may communicate better within the maternity providers’ team and with the women and her family. The training, quality improvement and leadership mentoring should enhance this synergistically. Improved processes in the hospital leading to timely access to caesarean section or operative vaginal delivery will reduce hypoxic-ischaemic events and thus reduce stillbirths and neonatal deaths. Improved management of foetal distress through change of position, re-hydration or interruption of contractions may reduce the need for operative interventions. In addition, immediate breastfeeding is encouraged also for women after caesarean section deliveries leading to improved bonding, nutrition and optimal growth and development in early childhood.


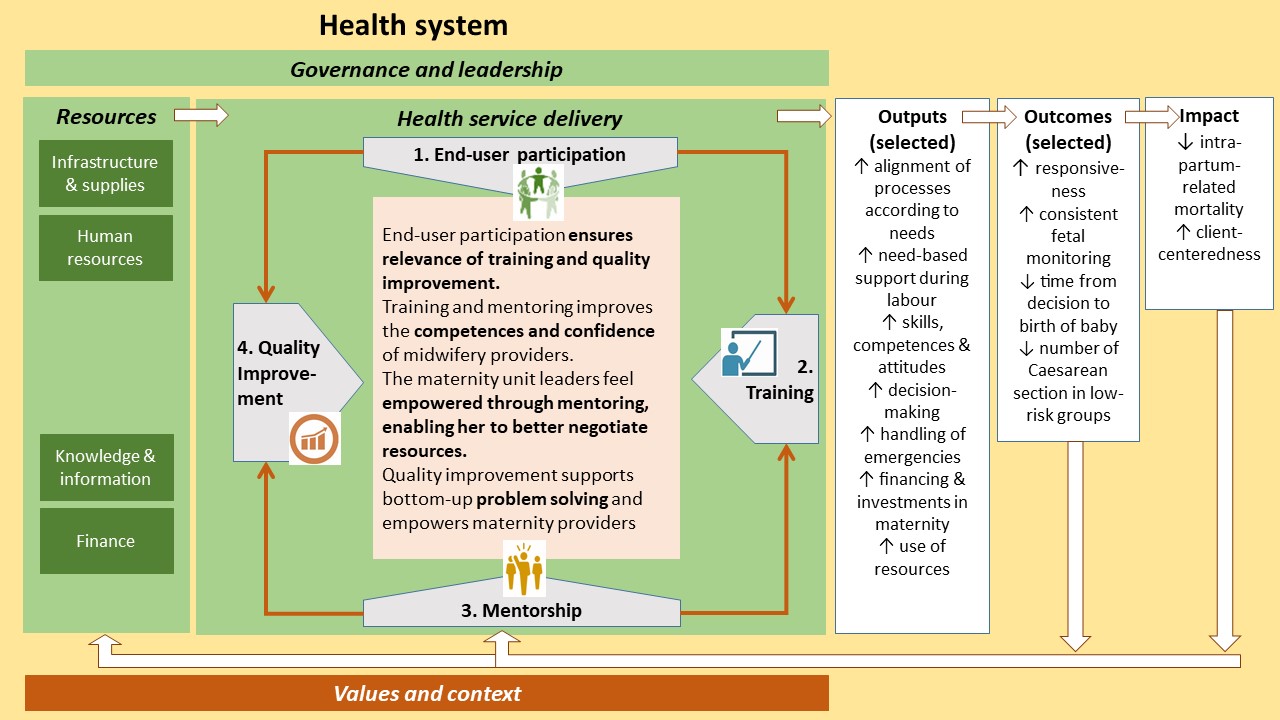


Figure 1: Initial Theory of Change


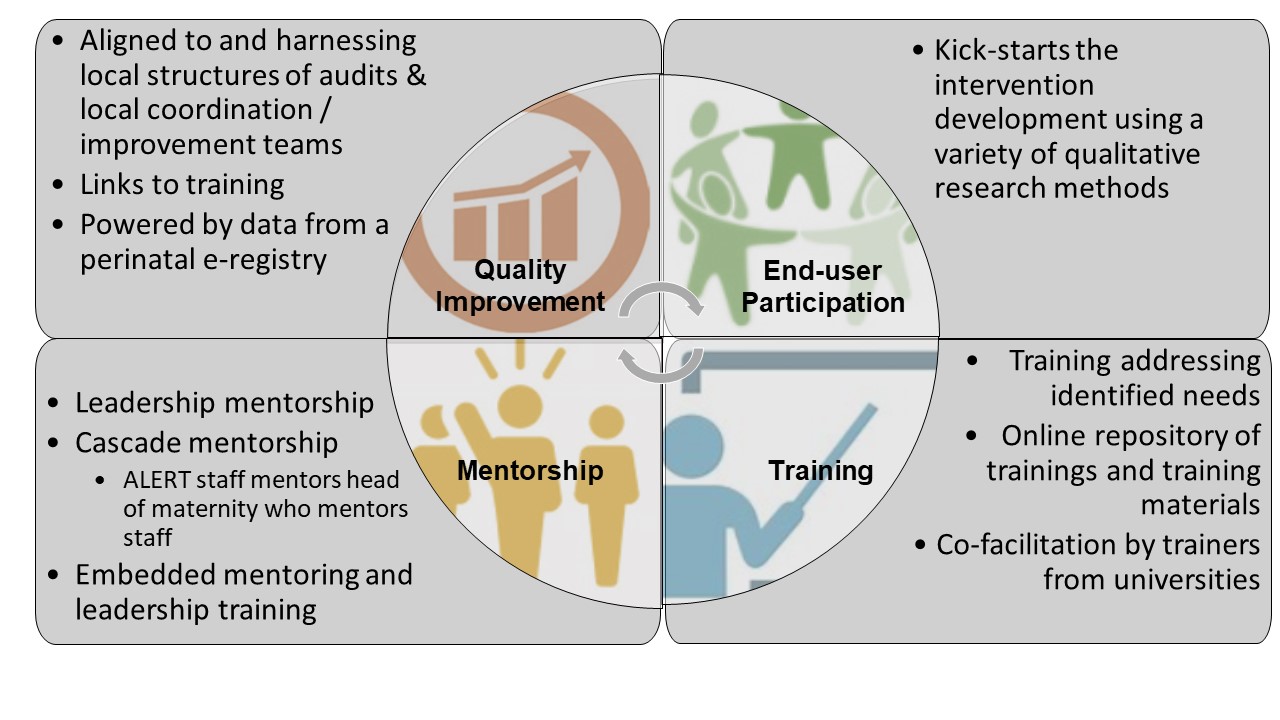


Figure 2: Synergies between the intervention components

#
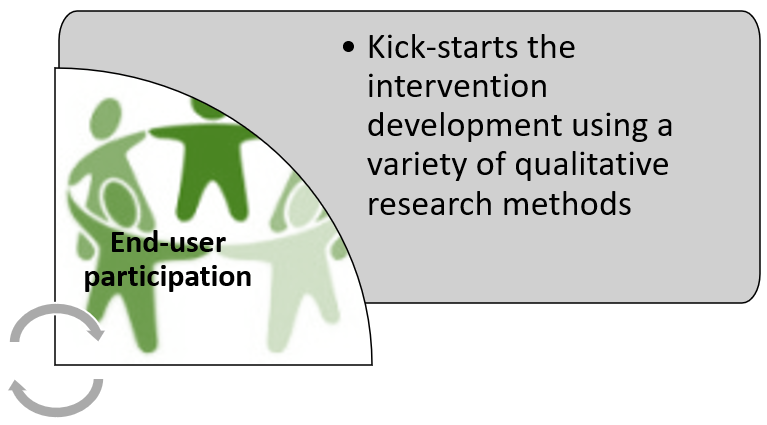
Tentative development of the intervention components:

1. **End-user participation**

End-user participation ensures relevance of the specific quality improvement activities that will be developed and implemented within the ALERT project. During the first formative phase, we will collect a diverse set of data, based on individual interviews, group discussion and observations of provider-patient interactions in the delivery ward as listed above. Based on a heterogeneity assessment of the 16 participating hospitals, two hospitals in each of the four countries are selected for in-depth data collection.

Data triangulation of the gender based multi-disciplinary labour wards through interviews with different midwifery providers will help us identify the barriers and facilitators of responsiveness and professionalism. In parallel, interviews and observations of interactions between women, their companions and midwifery providers will provide data on the diversity of ways in which communication and support is managed during the process of childbirth. By developing typologies of core moments in the care provision such as ways in which women are admitted to the hospital, roles of women’s companions, support during labour pain and fear, we will kick-off the process of co-designing the ALERT intervention.

The co-design approach aims to empower providers and patients with a stronger voice in improving healthcare quality. It involves borrowing from participatory and user experience design to bring quality improvement to healthcare organizations. After setting up the contact with the hospitals and the ALERT consortium and preliminary analysis of the collected qualitative data, we will gather midwifery providers and women who gave births at the hospital to:

1. discuss the preliminary findings;
2. identify priorities and
3. develop changes that are easy/difficult to implement on a short to medium term basis.

This co-design process, together with the other parts of the ALERT intervention package, should lead to better care processes for women and for providers to reconnect with their core values in midwifery care.

The data collected through the co-design approach will inform the ALERT intervention development. This approach will be followed:

1. We will disseminate co-design data to the ALERT consortium and map out a plan for intervention modification.
2. We will then work with individual teams to unpack the co-design data that relate to their work stream
3. Facilitate a workshop to review the refined intervention in terms of how well it has incorporated co-design recommendations.
4. ***Competency based training***
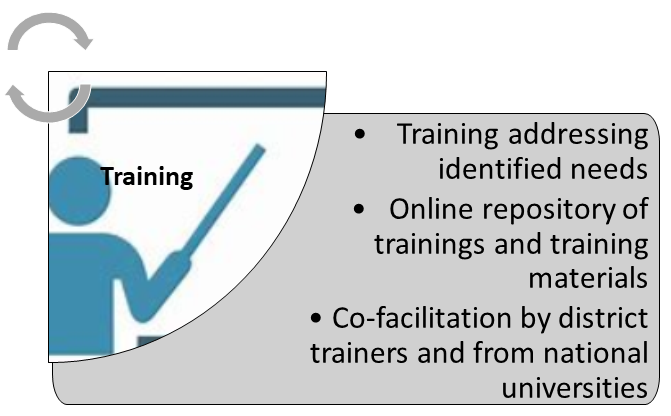


We will design competency-based training based on midwifery ^9,10,^ addressing aspects of intrapartum care. The training will be made available to all midwifery providers, regardless whether the primary education is in nurse-midwifery, midwifery, or medicine. The training will be implemented as in-facility training complemented by a tool-kit of self-learning materials. We aim to link to internet-based platforms, drawing on the ever-increasing list of online resources. We will use the MamaBirthie low-cost simulation material available from Laerdal Global Health.^11^ We will synergise our initiatives with other ongoing initiatives in the four countries including those implemented by WHO, Jhpiego and other partners, e.g. Laerdal Global Health, who are involved in training development. The ALERT consortium consists of leading universities in each of the project countries, which will allow our approach to be embedded into secondary and tertiary education in midwifery at Certificate, Bachelor, Masters and PhD levels in the four countries.

Evidence suggests training that includes a combination of lectures and the opportunity for simulation based participatory training has greater potential to improve team performance in terms of knowledge, skills and confidence, than lectures alone ^12^. The ALERT project proposes to follow this model for the delivery of the competency-based training package:

- Initial training sessions for midwifery care providers will provide theoretical knowledge and simulation opportunities.
- Clinical mentors will be identified and will facilitate low-dose high-frequency simulation opportunities in the hospital setting following the initial training ^13^.

Initial Suggestions for content include:

- Admission assessment
- Surveillance of labour: latent/first/second/third stage of labour
- Emergency preparedness: all the concepts that are taught regarding admission and surveillance of labour should include guidance on steps to be taken in the event of abnormal observations/progress of labour
- Labour companionship
- Respectful care: All the concepts that are taught regarding admission and surveillance of labour should include guidance on how to provide responsive, respectful care.

Delivering the training

Based on the findings of work undertaken on mentorship the following programme of delivery is suggested:

- Trainers
  - Will be certified and experienced trainers who are selected based on previous experience in training on maternal and child health within each country
  - Will be trained by ALERT team members in the content and delivery methods of the intrapartum training package
  - Will have access to the ALERT team for ongoing support throughout the duration of training
- Initial training session
- Led by certified and experienced trainers who have had training on the ALERT intrapartum training package
- Provides theoretical knowledge and opportunities for simulation of care
- Half day up to 2 days long
- Takes place on the hospital site
- Low-dose high-frequency simulation mentors
- Will be midwifery care providers from each hospital who are identified during the initial training session as competent with good communication and teaching skills
- Will have access to the trainers and the ALERT team for ongoing support throughout the duration of the Low-dose high-frequency simulation sessions.
- Follow up mentorship sessions
- Take place in the hospital once a week
- Provide an opportunity for the intrapartum training package skills to be practised in the clinical setting

1.
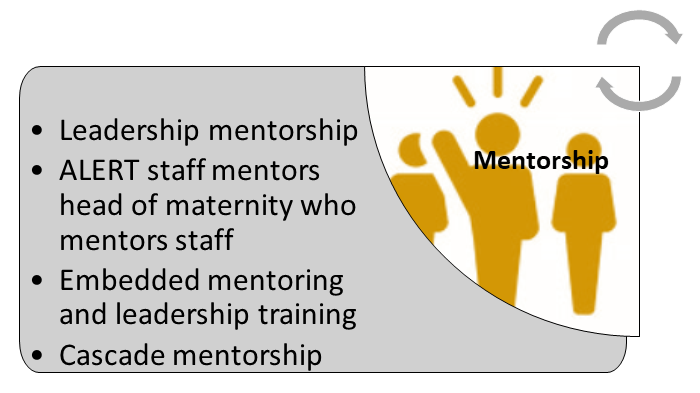
***Leadership mentorship***

Mentorship is increasingly recognised as an effective strategy to improve quality of health services, either as part of a QI bundle or as a stand-alone intervention ^14,15^. Gender aspects with their socio-cultural dimensions shape social attitudes and practices as well as work processes. Much too often, hospital and district budgets are decided by male district managers, while the task of reducing maternal and perinatal mortality is shouldered by female providers.^16^ Women make up 70% of the global workforce, yet only 25% of senior roles are held by women ^17^.

Such structural and operational misalignment will need to be identified and addressed. Those tasked with improvement need to have the control over resources ^18^

Improvement in intrapartum care requires strong leadership at all levels of the healthcare system, from the clinical bedside to organisational level. These leaders may benefit from mentorship. The aim of the leadership mentoring package is to support maternity unit leaders, who are predominately women, to provide supportive and effective leadership. Such leadership supports hospital teams to work effectively and address gaps that may be present in relation to the uptake of evidence-based practice.

The ALERT leadership mentoring plans to support the leadership skills of the maternity unit leaders by linking her/him with an experienced midwife from a national university included in the ALERT team. It is proposed that leadership training supports the maternity unit leaders to take transformative action as well as to support the facilitation of the competency-based training package. The leadership mentoring will integrate socio-cultural and gender dimensions. Access to resources is central to improvement. Leadership mentoring will commence as soon as possible after the data collection of the formative phase is completed, so that maternity unit leaders can be involved in developing and implementing the competency-based training package. Responsiveness and professionalism should be addressed through mentorship and suggestions for content of discussions/training include:

- Individual professional attitudes
- Skills to lobby for the needs within the maternity unit
- Accountability
- Inter-professional collaboration (teamwork)
- Mentorship to other team members
- Trust
- Provision of woman and family centred care
- Creation of partnerships with communities
- Leadership mentoring including resource negotiation skills.

Based on the findings of work undertaken on mentorship the following programme of delivery is suggested:

- Mentors
  - Will be midwives from a local national university who have clinical, academic and leadership experience, as well as good communication, teaching and problem solving skills
  - Will have access to the ALERT team for ongoing support throughout the duration of the leadership mentorship
- Leadership mentorship delivery
  - Initial educational sessions on aspects of leadership, mentorship and problem solving skills
  - Mentors and mentees will follow Plan, Do, Study, Act (PDSA) cycles to identify ‘change ideas’ and learning opportunities and techniques to facilitate the implementation of these ‘change ideas’
  - Monthly mentor visits to the facility to offer support to maternity unit leaders combined with phone calls as required

1.
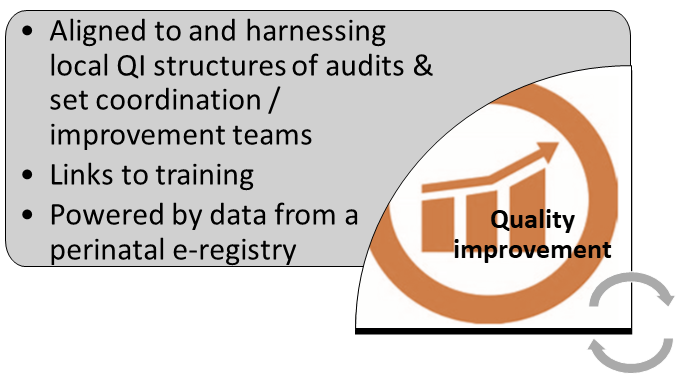
***Quality Improvement (QI)***

QI with their problem-solving approach have been identified as an important component of strategies to improve health workers’ action where human resources and supplies were not limiting implementation.^1^ QI is a key approach supported by the WHO and UNICEF as part of the multi-country initiative of the Quality, Equity, Dignity network.^19^ The approach has been used in several projects in Sub-Saharan Africa, albeit not always with major success.^4,20^ The Preterm Birth initiative, implemented in Kenya and Uganda, indicated the potential of QI implemented in combination with training.^3^ A recent review on supportive elements of the context and mechanisms of collaborative QI initiatives highlights the need to incorporate QI into a broader approach including leadership support and training.^21^

In addition to the quality improvement initiatives, maternal and perinatal death audits have been a key strategy to improve care around childbirth since the 1990s.^22^ The WHO supports the implementation of maternal and perinatal deaths audits as part of the Maternal death surveillance and response strategy.^23^ This strategy is also strongly backed by the professional association of Gynaecology and obstetrics.^24^ However, perinatal death audits and quality improvement strategies are not typically linked in facilities and linking these two strategies could have benefits.

Our preliminary review highlighted that death reviews and quality improvement are established approaches in all our implementation countries.

Table 1: Death review and quality improvement

|  |  | **Benin** | **Malawi** | **Tanzania** | **Uganda** |
| --- | --- | --- | --- | --- | --- |
| **Death reviews** | Maternal reviews | well established, ad-hoc within a few days after a maternal death | | | |
|  | Perinatal reviews | Yes | No | Not regular | Yes |
|  | Teams | Review committee between 10 to 17 members, including the head of maternity but limited formal and informal links to hospitals and district management committees | | | |
|  | Action plan / follow-up | Recommendation and proposed interventions are shared, albeit not well formalised and typically only reviewed within review team | | | |
| **Quality improvement** | Type of team | Quality improvement council | Quality improvement support team (QIST) and Work improvement team (WIT) | Work improvement team (WIT) | Quality improvement teams same as deaths review team |
|  | Functionality | monthly meetings | variable, monthly meetings | monthly meetings | monthly meetings, but variable functionability |
|  | Teams | Variable team sizes, depending on department/ward, head of maternity included in the maternity team | | | |
|  | Selection of improvement topics |  | Performance driven against national targets | Performance driven against national targets | Performance driven against national targets |
|  | Follow-up | Within the quality improvement teams, but variable | | | |

The aim of the QI package is to address malfunctioning processes within the ALERT study hospitals. Our approach will be mainly **based on the established quality improvement team in the maternity wards.**

Still, our approach will provide support to both the quality improvement teams and the death review committees. We will discuss with both these teams to locally find better ways to link the two quality improvement structures. The main strength of the death reviews is to point to specific problems and deficiencies and generate priorities, while quality improvement approaches, with their system of follow-up using clear indicators, can work more continuously and sustainably.

The **topics for the QI package** will be generated in response and aligned to

- the trainings provided to the midwifery care providers
- operational deficiencies identified during the formative research and as part of the end-user participation strategies
- the established deaths review teams
- hospital improvement topics

Our overall QI approach will be informed by the collaborative QI approach. We are aware that previous QI studies in sub-Saharan Africa have had limited effects. To overcome these and to maximize the effectiveness of our QI package, we plan to make adaptations to the collaborative QI approach as detailed in table 2. Clear aims will be generated, and indicators easy to monitor by the teams will be agreed on. The indicators should be available from case notes or the perinatal e-registry. The training sessions and the follow-up sessions should integrate the generation of so-called change ideas. Change ideas are proposed changes in processes or structures which are thought to impact the respective improvement goal. The overall goals of improving intrapartum care and reducing mortality will be monitored using the perinatal e-registry.

- Quality improvement teams will be supported to use Plan-Do-Study-Act (PDSA) cycles. Groups will follow PDSA cycles to understand whether “change ideas” result in improvement in hospital systems and adapt these ideas as required.
- Exchange with other quality improvement team within the facility and district will be supported.

| Table 2: Adaptations to the collaborative QI approach for the ALERT intervention | | | |
| --- | --- | --- | --- |
| Intervention feature | **IHI Breakthrough Collaborative Approach ^25-27^** | **Adaptation envisaged** | **Expected results** |
| Collaborative learning | A series of collaborative learning sessions, involving QI teams from each participating site. Learning sessions focus on the QI approach and lessons learned through implementation of PDSA cycles. | Due to the stepped wedge design of the ALERT intervention, mini collaboratives will develop over time with each hospital joining the collaborative learning sessions as they are enrolled to the intervention.  Maternity care providers in each facility will receive competency-based training which will be explicitly linked to QI. | The inclusion of several hospitals in the four countries will shape the attention to high level policy and structural barriers. |
| Quality improvement (QI) team composition | QI teams including health professionals from multiple professions are set up in each participating facility. | We will support the existing QI teams which have been established in all the four countries. Inclusion of QI in the competency-based training and the QI teams links together all members working in maternity. In addition, we aim to link to other established auditing and management structures. | The explicit link to existing QI structures is expected to increase the ease of implementation, improve sustainability and harness additional accountability structures. |
| Topic selection for QI | *Top-down selection of priorities:* The hospital selects an aim for improvement and implements PDSAs for that aim.  Common measures to assess performance and drive improvement, including sharing of data in the collaborative. | Mix of top-down and bottom-up selection of priorities.  Priorities should be set through 1) end-user participation, 2) links to established local perinatal auditing teams and 3) competency-based training sessions. | The end-user participation, competency-based training and the inclusion of perinatal audit findings should allow a mix of priorities to be set. These priorities are perceived locally as relevant but also as internationally prioritised and supported by literature. |
| Data for follow-up | Locally established data systems. | The perinatal e-registry will establish a standardised monitoring system which will allow the regular review of outcome and impact indicators. | The investment in a standardised data system should support exchange and accountability. |
| External support for QI | *Two mentors per facility:* a subject-matter expert (clinical expertise) and a quality improvement expert. | Cascade mentoring which focuses on support within the maternity teams as well as mentoring of the head of the maternity unit by midwives of national research institutions including leadership training. | We expect that the link to national staff will empower the head of maternity substantially through mentoring and leadership training. |

QI – Quality Improvement; PDSA – Plan-Do-Study-Act; IHI - Institute for Healthcare Improvement

**References**

1. Rowe AK, Rowe SY, Peters DH, Holloway KA, Chalker J, Ross-Degnan D. Effectiveness of strategies to improve health-care provider practices in low-income and middle-income countries: a systematic review. *The Lancet Global Health* 2018; **6**(11): e1163-e75.

2. Rowe AK, Labadie G, Jackson D, Vivas-Torrealba C, Simon J. Improving health worker performance: an ongoing challenge for meeting the sustainable development goals. *BMJ* 2018; **362**.

3. Walker D, Otieno P, Butrick E, et al. Effect of a quality improvement package for intrapartum and immediate newborn care on fresh stillbirth and neonatal mortality among preterm and low-birthweight babies in Kenya and Uganda: a cluster-randomised facility-based trial. *Lancet Glob Health* 2020; **8**(8): e1061-e70.

4. Waiswa P, Manzi F, Mbaruku G, et al. Effects of the EQUIP quasi-experimental study testing a collaborative quality improvement approach for maternal and newborn health care in Tanzania and Uganda. *Implementation Science* 2017; **12**(1): 89.

5. Baker U, Petro A, Marchant T, et al. Health workers' experiences of collaborative quality improvement for maternal and newborn care in rural Tanzanian health facilities: A process evaluation using the integrated 'Promoting Action on Research Implementation in Health Services' framework. *PLOS ONE* 2018; **13**(12): e0209092.

6. Zamboni K, Singh S, Tyagi M, Hill Z, Hanson C, Schellenberg J. Effect of collaborative quality improvement on stillbirths, neonatal mortality and newborn care practices in hospitals of Telangana and Andhra Pradesh, India: evidence from a quasi-experimental mixed-methods study. *Implement Sci* 2021; **16**(1): 4.

7. Kruk ME, Gage AD, Joseph NT, Danaei G, García-Saisó S, Salomon JA. Mortality due to low-quality health systems in the universal health coverage era: a systematic analysis of amenable deaths in 137 countries. *The Lancet* 2018; **392**(10160): 2203-12.

8. Roder-DeWan S, Nimako K, Twum-Danso NAY, Amatya A, Langer A, Kruk M. Health system redesign for maternal and newborn survival: rethinking care models to close the global equity gap. *BMJ Global Health* 2020; **5**(10): e002539.

9. Renfrew MJ, McFadden A, Bastos MH, et al. Midwifery and quality care: findings from a new evidence-informed framework for maternal and newborn care. *The Lancet* 2014; **384**(9948): 1129-45.

10. FIGO, IMBCO, partners. The International Childbirth Initiative (ICI): 12 Steps to Safe and Respectful MotherBaby-Family Maternity Care. 2018.

11. Laerdal Medical. MamaBirthie. <https://laerdal.com/se/products/simulation-training/obstetrics-paediatrics/mamabirthie/> (accessed 18 Feb 2021).

12. Ameh CA, Mdegela M, White S, van den Broek N. The effectiveness of training in emergency obstetric care: a systematic literature review. *Health Policy and Planning* 2019; **34**(4): 257-70.

13. Alwy Al-beity F, Pembe A, Hirose A, et al. Effect of the competency-based Helping Mothers Survive Bleeding after Birth (HMS BAB) training on maternal morbidity: a cluster-randomised trial in 20 districts in Tanzania. *BMJ Global Health* 2019; **4**(2): e001214.

14. Magge H, Anatole M, Cyamatare FR, et al. Mentoring and quality improvement strengthen integrated management of childhood illness implementation in rural Rwanda. *Arch Dis Child* 2015; **100**(6): 565-70.

15. Manzi A, Magge H, Hedt-Gauthier BL, et al. Clinical mentorship to improve pediatric quality of care at the health centers in rural Rwanda: a qualitative study of perceptions and acceptability of health care workers. *BMC Health Serv Res* 2014; **14**: 275.

16. World Health Organization, Global Health Workforce Network, Women in Global Health. Delivered by women, led by men: a gendered and equity analysis of the global health and socail workforce. Geneva, 2019.

17. World Health Organization, Global Health Workforce Network, Women in Global Health. Delivered by women, led by men: a gender and equity analysis of the global health and social workforce. 2019. <https://apps.who.int/iris/bitstream/handle/10665/311322/9789241515467-eng.pdf> (accessed 5 Jan 2020).

18. Coret A, Boyd K, Hobbs K, Zazulak J, McConnell M. Patient Narratives as a Teaching Tool: A Pilot Study of First-Year Medical Students and Patient Educators Affected by Intellectual/Developmental Disabilities. *Teaching and Learning in Medicine* 2018; **30**(3): 317-27.

19. World Health Organization. Quality, Equity, Dignity: the network to improve quality of care for maternal, newborn and child health 2018. <https://www.who.int/maternal_child_adolescent/documents/quality-care-network-objectives/en/> (accessed 28 Nov 2020).

20. Larson E, Mbaruku GM, Cohen J, Kruk ME. Did a quality improvement intervention improve quality of maternal health care? Implementation evaluation from a cluster-randomized controlled study. *International Journal for Quality in Health Care* 2019; **32**(1): 54-63.

21. Zamboni K, Baker U, Tyagi M, Schellenberg J, Hill Z, Hanson C. How and under what circumstances do quality improvement collaboratives lead to better outcomes? A systematic review. *Implementation Science* 2020; **15**(1): 27.

22. AbouZahr C, De Bernis L, Guidotti R, Van Look P, Zupan J. Beyond the numbers. Reviewing maternal deaths and complications to make pregnancy safer. Geneva; 2004.

23. WHO, FIGO, UKaid, et al. Maternal death surveillance and response. Technical guidance. Information for action to prevent maternal death. 2013. <http://apps.who.int/iris/bitstream/10665/87340/1/9789241506083_eng.pdf?ua=1>.

24. De Brouwere V, Zinnen V, Delvaux T, Nana PN, Leke R. Training health professionals in conducting maternal death reviews. *International Journal of Gynecology & Obstetrics* 2014; **127**(S1): S24-S8.

25. Institute for Healthcare Improvement. The Breakthrough Series: IHI’s Collaborative Model for Achieving Breakthrough Improvement. IHI Innovation Series white paper. Boston: Institute for Healthcare Improvement, 2003.

26. Kilo CM. A framework for collaborative improvement: lessons from the Institute for Healthcare Improvement's Breakthrough Series. *Qual Manag Health Care* 1998; **6**(4): 1-13.

27. Wilson T, Berwick DM, Cleary PD. What do collaborative improvement projects do? Experience from seven countries. *The Joint Commission Journal on Quality and Patient Safety* 2003; **29**(2): 85-93.
