# Supplementary figures and images for: Action leveraging evidence to reduce perinatal mortality and morbidity (ALERT): study protocol for a stepped-wedge cluster-randomised trial in Benin, Malawi, Tanzania and Uganda
Source: BMC Health Serv Res. 2021 Dec 11;21:1324. doi: 10.1186/s12913-021-07155-z (PMC8665312; doi:10.1186/s12913-021-07155-z)

**Additional file 4: Sample size calculation (ICC, Inter Cluster Coefficient)**


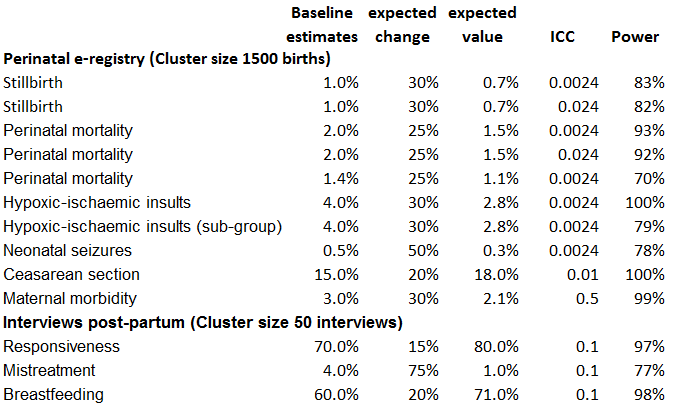

Supplement: Supplementary file 4 — Additional file 4. Sample size calculation (ICC, Inter Cluster Coefficient). [file 12913_2021_7155_MOESM4_ESM.docx]
